# Early-life stress and life

**DOI:** 10.18632/aging.101568

**Published:** 2018-09-23

**Authors:** Yusuke Takatsuru

**Affiliations:** 1 Department of Medicine, Johmoh Hospital, Maebashi, Gunma 379-2152, Japan

**Keywords:** maternal deprivation, aging, multie generation effect, dopmanie, prolactin

Early-life stress during childhood persistently impairs cognitive and emotional functions until adulthood in humans. However, it has not yet been clarified how the type, intensity and duration of stress affect different brain regions with different persistence [1]. To clarify the mechanisms underlying the effects of early-life stress on brain function, I and colleagues use maternal deprivation (MD) mice and reported that homeostasis of glutamate release is disrupted, and the change induces instability of spines, hyperactivity of neurons, and hypersensitivity [2,3].

**Figure 1:**
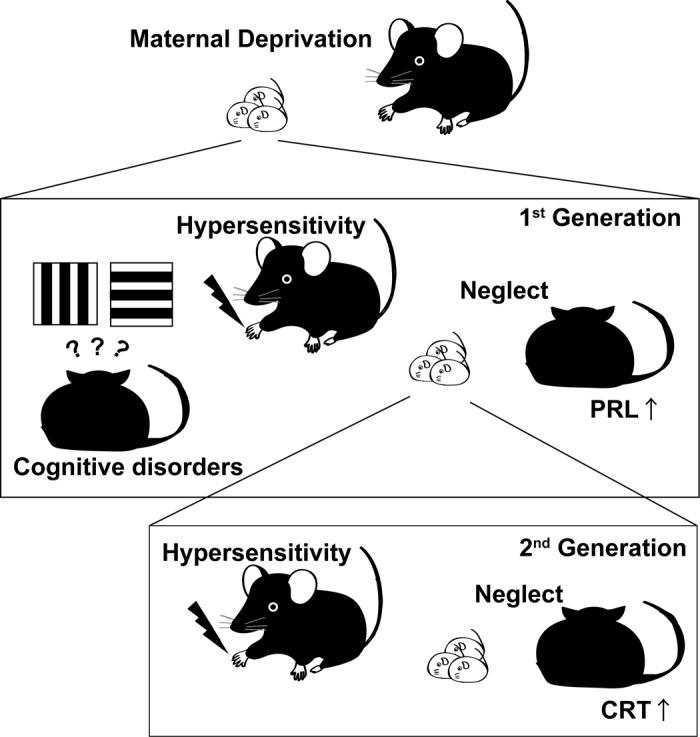
**“Super” chronic effects of maternal deprivation on brain function and behavior of mice.** Early-life stress induces hypersensitivity, early-onset of cognitive function disorder, and neglect behavior of mothers. Early-life stress also induces the increase in the concentration of prolactin (PRL) during the perinatal period. Which potentially increases the concentration of corticosterone (CRT) in offspring. These changes possibly induce hypersensitivity and neglect behavior of offspring.

Early-onset dementia could be one of the social demerits if we are thinking about family life. People in their 30 - 40s are the main earners. They also mainly play a critical role in rearing their kids. If a parent suffers from dementia and is unable to work, this poses difficulties not only for the parent but also the whole family including the next generation (it could also be a new risk factor for early-life stress). We recently reported the retardation of cognitive function in middle-aged MD mice (approximately 1.4 years old) ([4]. See also the Figure 1). We also found that the decrease in correct response rate in middle-aged MD mice was not induced by “difficulty in continuing the task (because of poor motor function, depressive behavior, and/or attention/activity disorders)”. These results also indicate that middle-aged mice at least became habituated and learned how to continue the task. However, it cannot be concluded that the middle-aged MD mice have no problems in terms of their mental/physical state. Further study will be required to observe the emotional/ cognitive function of middle-aged MD mice. If we consider early-life stress as a risk factor for early-onset dementia, clarifying the mechanisms by which this factor functions by studying of MD mice, may contribute to the social wellbeing of affected individuals.

Furthermore, we have recently reported that early-life stress induces neglect behavior of early-life-stressed mothers, which was also detected in the offspring of early-life-stressed mothers ([5]. See also the Figure 1). This finding indicates that early-life stress induces severe effects not only the individual directly exposed to stress but also its offspring. A recent study in humans showed that a victim of childhood abuse tends to abuse their offspring [6]. However, not all victims become abusive. Additional genetic/epigenetic factors may be required to produce abnormal behavioral alterations in the next generation. Indeed, our previous study, several parameters examined did not show statistically significant changes owing to large variances, indicating individual variations of maternal influences ([5] and unpublished data). Environmental conditions during and after lactation [relationships with other mice living in the same cage, temperature/humidity (even under controlled condition), atmosphere (which we unable to control), smell, sound, and/or humans (access to the animal room, smell of humans)] may also modulate the brain development of the offspring of early-life-stressed mothers. Such additional environmental factors affect the generation/regeneration of neuronal circuits. PRL and dopamine are strong candidate factors whose mechanisms of inducing early-life stress and effects on brain function should be clarified. On the other hand, how these internal factors contribution to is very complicated. Both too low [7] and too high [5] concentrations of PRL adversely affect maternal behavior. Therefore, if we are thinking of drug treatment, moderate control is necessary.

A rodent model is useful for chronic, multigeneration studies. However, “a rodent is a rodent”. In the medical field, studies on humans are also necessary to clarify the mechanisms by which early-life stress affects brain function. To study the chronic effect of stress, *in vivo* whole-animal studies are the most important approach to consider. However, progress in such studies is insufficient because of technical limitations (e.g., we cannot confirm functional, structural, and molecular changes several times in the “same” animal). Technical innovations will also be necessary in the future and some of which may not be achieved very soon.
